# Kerosene Lamps

**Published:** 1877-04

**Authors:** 


Kerosene Lamps.—The Wiener Medicinische Presse says: " A merchant returned home about two o'clock at night, and found his wlfe lying on the bed, groaning heavily, and unconscious. She was waiting his return, and at last, tired out, laid herself upon the bed, after turning down the wick of a lighted kerosene lamp as low as possible without extinguishing it. In this position of the wick, if the oil is bad, a vapor mixed with an innumerable quantity of specks of soot diffuses itself through the apartment, and so covers the eyes, nose, and respiratory organs that on falling asleep one runs a risk of suffocation. It is always advisable, therefore, in the use of kerosene lamps to allow the wick to burn brightly or to extinguish it entirely." A physician in Kentucky traced without a doubt his attacks of asthma to the use of kerosene lamps.—*Louisville Md. News.*

